# Predicting language recovery in post-stroke aphasia using behavior and functional MRI

**DOI:** 10.1038/s41598-021-88022-z

**Published:** 2021-04-19

**Authors:** Michael Iorga, James Higgins, David Caplan, Richard Zinbarg, Swathi Kiran, Cynthia K. Thompson, Brenda Rapp, Todd B. Parrish

**Affiliations:** 1 Center for the Neurobiology of Language Recovery, Northwestern University, Evanston, IL USA; 2 Department of Biomedical Engineering, McCormick School of Engineering, Northwestern University, Chicago, IL USA; 3 Department of Radiology, Feinberg School of Medicine, Northwestern University, Suite 1600, 737 N. Michigan Ave., Chicago, IL 60611 USA; 4 Department of Neurology, Massachusetts General Hospital, Harvard Medical School, Boston, MA USA; 5 Department of Psychology, Northwestern University, Evanston, IL USA; 6 Department of Speech, Language, and Hearing, College of Health and Rehabilitation, Boston University, Boston, MA USA; 7 Department of Communication Sciences and Disorders, School of Communication, Northwestern University, Evanston, IL USA; 8 Department of Neurology, Neurology, Feinberg School of Medicine, Northwestern University, Chicago, IL USA; 9 Department of Cognitive Science, Krieger School of Arts and Sciences, Johns Hopkins University, Baltimore, MD USA

**Keywords:** Neuroscience, Computational neuroscience, Diseases of the nervous system, Computational models, Computational neuroscience, Image processing

## Abstract

Language outcomes after speech and language therapy in post-stroke aphasia are challenging to predict. This study examines behavioral language measures and resting state fMRI (rsfMRI) as predictors of treatment outcome. Fifty-seven patients with chronic aphasia were recruited and treated for one of three aphasia impairments: anomia, agrammatism, or dysgraphia. Treatment effect was measured by performance on a treatment-specific language measure, assessed before and after three months of language therapy. Each patient also underwent an additional 27 language assessments and a rsfMRI scan at baseline. Patient scans were decomposed into 20 components by group independent component analysis, and the fractional amplitude of low-frequency fluctuations (fALFF) was calculated for each component time series. Post-treatment performance was modelled with elastic net regression, using pre-treatment performance and either behavioral language measures or fALFF imaging predictors. Analysis showed strong performance for behavioral measures in anomia (R^2^ = 0.948, n = 28) and for fALFF predictors in agrammatism (R^2^ = 0.876, n = 11) and dysgraphia (R^2^ = 0.822, n = 18). Models of language outcomes after treatment trained using rsfMRI features may outperform models trained using behavioral language measures in some patient populations. This suggests that rsfMRI may have prognostic value for aphasia therapy outcomes.

## Introduction

Aphasia is an acquired impairment in language production and/or comprehension which manifests in one third of stroke survivors^1–3^. Post-stroke aphasia is managed with speech and language therapy (SLT), which addresses language impairments through patient-specific, targeted training in order to improve functional communication^4,5^. Despite strong evidence that SLT is an effective therapy for post-stroke aphasia, a majority of patients with acute aphasia will continue to experience aphasia chronically^6,7^. As aphasia significantly lowers functional independence and health-related quality of life, there is a need to improve the currently available therapeutic options for post-stroke aphasia^2,8,9^. While the efficacy of alternatives to and variations of SLT has been investigated, there is currently not enough evidence to recommend one therapy over another^4^. Improving aphasia therapy is difficult due to high variability in patient response: some patients fully recover while others experience little benefit^10–13^. Overcoming this variability in response may be possible by personalizing aphasia therapy, however the precise relationships between patient-level factors and recovery trajectories are not currently known^14^.

It has been shown that aphasia impairment, aphasia severity, stroke lesion location, and stroke lesion volume all influence a patient’s response to therapy^9,15^. Attempts at modelling recovery trajectories have therefore focused on interpreting these variables, among other patient-level factors. For example, one study modeled patient performance on the Aphasia Severity Rating Scale after one year of SLT using measures of language impairment, functional disability, age, education, and stroke type (R^2^ = 0.56, n = 147)^16^. Another study modeled improvements on a composite score of comprehension, repetition, and naming measures at two weeks after stroke using baseline performance on these measures as well as the lesion load, volume, and diffusion metrics (R^2^ = 0.73, n = 20)^17^. While these models capture most of the variance in functional outcomes, further improvements could be realized through an enhanced quantitative description of the baseline aphasia profile. To capture the impact of lesion distribution on specific functional deficits, Halai et. al. developed a model which considers the overlap of lesions with known core language areas to predict individual patient performance on a battery of 21 aphasia measures (mean R^2^ = 0.48, n = 70)^18^. This and earlier work suggest that a profile of aphasia impairments and severity can be inferred from lesion location and extent at the patient-level^19,20^. However there remains considerable unexplained variance in patient outcomes, and prognostic model performance may benefit from inclusion of additional functional variables^21,22^.

Functional neuroimaging has been applied extensively to assess and further understand the neural underpinnings of aphasia. Models which interpret neuropsychological measures alongside data-driven and/or multi-modal neuroimaging features have been successful in assessing aphasia severity^23–26^. Resting-state functional MRI (rsfMRI) has demonstrated particular potential as an aphasia assessment tool. Patients with aphasia can be distinguished from healthy controls by measuring differences in functional connectivity of resting networks^27–29^. Resting network activity can be used to infer the aphasia severity profile, and changes in global connectivity track the extent of language recovery^30–33^. These findings suggest that rsfMRI may serve as a complementary tool to traditional behavioral and anatomical assessments of aphasia. Models which predict patient response to aphasia therapy may therefore benefit from inclusion of functional imaging features^18^.

In this study, we examine the extent to which rsfMRI can predict aphasia severity after SLT, as compared to conventional behavioral measures. We first establish the baseline performance of prognostic models using a large battery of behavioral measures across three aphasia impairments: anomia, agrammatism, and dysgraphia. We next develop a second set of models for each impairment, using baseline aphasia severity and data-driven rsfMRI features. The performance of these prognostic models is discussed relative to models using only language and cognitive measures as predictors, as well as to prior work.

## Methods

### Recruitment and assessment

Patients with chronic aphasia were recruited from the Aphasia Research Laboratory at Boston University (BU), the Aphasia and Neurolinguistics Laboratory at Northwestern University (NU), and the Cognitive and Brain Sciences Laboratory at Johns Hopkins University (JHU). Patients were independently recruited, diagnosed, and treated for one aphasia impairment at each site: anomia at BU (N = 28), agrammatism at NU (N = 11), and dysgraphia at JHU (N = 18). The diagnosis of aphasia was made using the mean score on the Western Aphasia Battery revised (WAB-R)^34^. All patients presented with aphasia resulting from a single left-hemisphere thromboembolic or hemorrhagic stroke (see Supplementary Fig. S1 for the aggregate lesion map), were at least one-year post-stroke, and had no other impairments that impacted the ability to complete the behavioral or neural tasks (e.g. vision and hearing was within normal limits). All were monolingual English-speaking, had at least a high school education, and completed a written consent form approved by every site’s Institutional Review Board (IRB). All patients included in this study were right-handed, as determined through the Edinburgh Handedness Inventory. All experiments and protocols described in the upcoming sections were performed in accordance with the guidelines and regulations put forth by the IRB from each participating institution.

Aphasia is primarily diagnosed and assessed through language assessments, such as the Western Aphasia Battery (WAB)^34^. However, the WAB lacks sensitivity to lexical-semantic deficits, sentence processing deficits, and spelling deficits, so supplementary language measures must also be performed to capture the full range of aphasic deficits^35–37^. While many language measures have been developed to test specific language deficits, relatively few have been assessed for psychometric validity^38–41^. As a result, there is a lack of consensus on the optimal aphasia assessment battery, and the prognostic utility of existing language measures are largely unknown. In order to be inclusive of potentially prognostic measures, we collected a broad range of 27 behavioral measures from eleven language and cognitive assessments (see Table 1). Each patient was also assessed on one of three treatment-specific measures (TSMs, defined below), which served as the primary metric for evaluating the baseline aphasia severity and the response to SLT. Patients also underwent comprehensive multi-modal imaging assessment, including T1 structural MRI, perfusion, diffusion, task-based and resting-state fMRI. The present study only examines the rsfMRI data.

**Table 1:** Aphasia assessment battery.

Assessment (acronym)	Measure (acronym)
Western Aphasia Battery (WAB)^34^	Information Content (IC) Fluency (FL) Comprehension (CO) Repetition (RE) Naming (NA)
Northwestern Naming Battery (NNB)^72^	Noun Comprehension (NC) Verb Comprehension (VC) Noun Production (NP) Verb Production (VP)
Northwestern Assessment of Verbs and Sentences (NAVS)^73^	Canonical Sentence Comprehension Test (SCT-C) Noncanonical Sentence Comprehension Test (SCT-N) Canonical Sentence Production Priming Test (SPPT-C) Noncanonical Sentence Production Priming Test (SPPT-N)
Psycholinguistic assessments of language processing in aphasia (PALPA) 1^74^	Phonological Discrimination
PALPA 35	Reading Regular (RE) Reading Exception (EX)
PALPA 40	Spelling High Frequency Words (HF) Spelling Low Frequency Words (LF)
PALPA 51	Semantic Association: High Imageability (HI) Semantic Association: Low Imageability (LI)
Pyramids & Palm Trees (PPT)^75^	Semantic Association
Doors & People (D&P)^76^	Explicit Memory
Cinderella Story (CIND)^77^	Words per Minute (WPM) Mean Length of Utterance—Words (MLW) Mean Length of Utterance—Morphemes (MLM)
Digit Span (DS)	Forwards (FOR) Backwards (BAC)
Treatment-specific Measure (TSM)	Object Naming *or* Sentence Comprehension & Production *or* Spelling Words

All 27 behavioral measures comprising 11 language and cognitive assessments are shown. Each measure has a corresponding acronym constructed by hyphenating the assessment acronym with the individual measure acronym (i.e. the WAB-IC correspond to the Western Aphasia Battery, Information Content), except for measures in the NAVS which are referred to only by their measure acronym.

### Speech and language therapy

Following baseline testing, each patient received a three-month course of SLT. The TSM was measured both before and after completion of the treatment protocol to estimate the treatment response. Detailed treatment protocols have been described previously and are summarized here.

Patients with anomia underwent a typicality-based semantic treatment^42^. Each patient participated in a computer-based task where they were presented with pictures from five semantic categories: birds, vegetables, fruit, clothing, and furniture. Patients sorted images into categories, then attempting to name each image. Naming was confirmed using written and auditory verification, before a second naming attempt was made. Training occurred weekly and each patient was assigned two half-categories for review. Assessment of treatment progress was made using a comprehensive naming test of items from 3 of the five categories (Anomia TSM).

Patients with agrammatism received sentence comprehension and production treatment through a Treatment of Underlying Forms program^43^. In this treatment the patient was shown an action picture that depicts a scene. The patient was then given a set of cards with verbs, and asked to point to the action verb that describes that scene. The examiner then built an active verb sentence using the action verb card and an active sentence template, and demonstrated to the patient how the active sentence can be changed into a passive verb sentence. Next, the patient formed the passive sentence and read it aloud. This treatment occurred twice per week for 90 min per session. Assessment of treatment progress was made using a sentence production test. Patients were shown two action pictures, and given a sentence describe one of them. The patient then had to produce a similar sentence for the other picture. The produced sentences were recorded and subsequently assessed for semantic similarity to the prompt sentence (Agrammatism TSM).

Patients with dysgraphia underwent a spell-study-spell treatment protocol^44^. Each patient was given a set of 40 training words to learn to spell. Word sets were customized by patient such that each word has a baseline letter accuracy between 25 and 85%. Treatment consisted of a patient hearing the word, repeating it, and attempting to write it out. This was repeated until the word was spelled correctly, up to a maximum of three attempts per word. The patient was shown the correct spelling of the word regardless of accuracy. Training occurred twice per week for 90 min per session. Assessment of treatment progress was made by measuring the letter accuracy across the entire training set (Dysgraphia TSM).

### Image acquisition

MRI scans were acquired using 3.0 T scanners (Siemens Skyra at BU, Siemens Trio/Prisma at NU, and Philips Intera at JHU). Imaging protocols were harmonized across the sites to provide similar quality and timing. Structural images were collected using a 3D T1-weighted sequence (TR = 2300 ms, TE = 2.91 ms, flip angle = 9°, resolution = 1 mm^3^ isotropic). Whole brain functional images were collected using a gradient-echo T2*-weighted sequence (TR = 2 or 2.4 s, TE = 20 ms, flip angle = 90°, resolution = 1.72 × 1.72 × 3 mm, 210 or 175 volumes). Initial studies (first 5 NU subjects) used a 2 s TR, but additional coverage was required to obtain whole brain data so TR was increased to 2.4 s. While NU and BU had one scan of 210 volumes, JHU subjects received 2 runs of 175 volumes each, and only the scan with the highest temporal signal-to-noise ratio (tSNR) was included for analysis.

### Image preprocessing

All images were archived on NUNDA (Northwestern University Neuroimaging Data Archive, https://nunda.northwestern.edu) for storage and data analysis. Upon arrival in the archive, image quality assurance (QA) was performed using automatic pipelines for functional and structural data. For fMRI data, a slice-wise tSNR was calculated as the ratio of the mean signal to the standard deviation of the time course data from each slice, weighted by the number of brain voxels in the slice. Poor-quality scans (tSNR < 100) were repeated or excluded from analysis.

Image preprocessing was performed using the NUNDA “Robust fMRI preprocessing pipeline”, which employs custom scripts built upon functions from AFNI, FSL, and SPM software^45–47^. First, the fMRI time series were despiked (AFNI 3dDespike) and coregistered to the mean image (AFNI 3dvolreg). Normalization to standard MNI space was performed in a concatenated two-step procedure. A transformation aligning the first image in the fMRI time series to the T1 was created using boundary based registration (FSL BBR)^48^. This was combined with the nonlinear warp of the T1 to an MNI template of 2 × 2 × 2mm resolution (SPM Dartel Toolbox)^49^. Structural images were corrected using enantiomorphic lesion transplant (SPM Clinical Toolbox) to minimize distortion effects caused by warping brains with lesions^50,51^. Using the lesion mask as a reference, right hemisphere homologous tissue was mirrored into the lesioned space to create a lesion-corrected left hemisphere. The optimal transform, calculated using the lesion-corrected brain, was then applied to the native brain.

### rsfMRI analysis

The rsfMRI features which predict aphasia recovery are currently unknown, encouraging a data-driven approach. Group independent component analysis (GICA) is a data-driven method of decomposing signals into underlying spatial and temporal components. Applied to rsfMRI, GICA may identify statistically independent patterns of brain activity across subjects. These patterns may then be compared across subjects, permitting analysis of network activity within and across groups. GICA also offers intrinsic noise filtering, as noise which is statistically independent of the signal filters into its own component. These components can be filtered out to perform group artifact removal, which has been demonstrated to be more reliable than artifact removal at the subject level^52^. Subjects from all sites were processed together to attenuate the impact of single-scanner artifacts on final components.

GICA was performed through the GIFT toolbox for MATLAB^53^. Data were decomposed using default parameters (20 components, InfoMax algorithm). Component projections clustered strongly across 100 random parameter initializations, indicating the chosen parameters were stable in our analysis^54^. GICA components were then backprojected, producing 20 spatial components and 20 corresponding time series for each subject. Component maps which have been aggregated across subjects are displayed in Supplementary Fig. S3.

Measurement of low-frequency oscillations is broadly used as a generalized activity metric in fMRI analyses, including studies in both stroke^55,56^ and aphasia^57,58^. The fractional amplitude of low-frequency fluctuations (fALFF) is the ratio of power in the 0.01–0.08 Hz band to the total power^59^. The fALFF was calculated for each time series of each component for each subject, then standardized such that the sum of a subject’s 20 fALFF values equals one. This permits comparison of relative component power within a subject, and normalizes activity ranges prior to regression analyses. We combined fALFF with GICA as a data reduction technique, whereby a series of functional volumes is summarized by an activity measure of 20 components. This approach helps avoid overfitting by reducing problem dimensionality, and makes regression more feasible with the given sample sizes.

### Model construction and validation

Prediction of post-treatment primary dependent measures was modeled using elastic net regression. This model was chosen because linear models are relatively robust to lower sample sizes, and tend to generalize well when trained with regularization. Elastic net regression utilizes a combination of LASSO (L1-norm) and ridge (L2-norm) regularization penalties, and reduces overfitting by limiting coefficient magnitudes^60^. Regularization hyperparameters were determined by leave-one-out cross-validation (LOOCV). To facilitate equitable interpretation of model coefficients, all input variables underwent z-score normalization prior to training. Model training and validation was performed with the caret package in R. Missing data were imputed using the randomForest package in R^61^. Imputation hyperparameters were selected to minimize output variance. All results from subsequent analyses with missing data were repeated using data from 1000 imputations, and the summary statistics across imputations are shown for each.

Model performance was assessed by comparing the post-treatment TSM predicted through LOOCV versus the actual post-treatment TSM. If the model predicted a value outside of the known range of a TSM (i.e. over 100% or below 0% accuracy), the result was rounded to within the test’s dynamic range. We measured error in model predictions using the median absolute deviation (MAD) between predicted and actual values. The MADs of each model were compared to the MADs between predicted post-treatment TSMs and their mean (zero-order model) using a paired Wilcoxon test to assess if the model performed better than chance. This nonparametric approach was taken due to non-normality and heteroscedasticity in the dataset. Second, we measured the percent of variability in post-treatment TSMs explained by each model using the square of the Pearson correlation coefficient (R^2^). We then tested if each correlation value is significantly larger than zero using a correlation coefficient hypothesis test.

### Ethics approval and consent to participate

All participants provided written informed consent according to Institutional Review Board policies at Boston University, Johns Hopkins University, and Northwestern university. All experiments in this study were performed in accordance with the guidelines and regulations put forth by these Institutional Review Boards.

## Results

### Participants

Demographics and aphasia severity for the 57 recruited participants are shown in Table 2. Of the participants who entered the study, one dropped immediately and one passed away before completing testing. Both of these participants were completely excluded from analyses. Of the remaining participants, one dropped out and one suffered a hematoma during therapy. For these participants the baseline language measures were used, and the post-treatment dependent measures were imputed. In addition, two agrammatism participants were scanned with a different sequence, and these subjects were removed from fALFF-based analyses. All baseline TSM data were collected, and 3.3% of the other baseline language measurements were missing due to incomplete testing and/or lack of follow-up. Patient performance on the TSM increased significantly over the course of treatment for all therapy groups: Anomia (*p* = 2.8E−6), Agrammatism (*p* = 0.005), Dysgraphia (*p* = 1.0E−4) (one-sided Wilcoxon Signed Rank Test).

**Table 2:** Subject demographics and WABAQ by language-specific deficit.

Attribute	Anomia (N = 28)	Agrammatism (N = 11)	Dysgraphia (N = 18)	*p*
Gender	F: 9 M: 19	F: 4 M: 7	F: 6 M: 12	1.000
Age	63.5 ± 10.4	51.0 ± 3.0	62 ± 11.1	**0.008**
Education (Years)	16 ± 1.5	18 ± 1.5	16 ± 3.0	**0.004**
months post stroke	27.5 ± 23.0	39 ± 28.2	52.5 ± 38.5	0.283
Aphasia severity (WABAQ)	62.2 ± 31.4	72.0 ± 17.3	86.8 ± 15.5	**0.018**

Counts by attribute and aphasia impairment are shown for categorical variables. Categorical variable *p* values are calculated with a two-sided Fisher’s Exact Test. For continuous variables, median ± median absolute deviation is shown. Continuous variable *p* values are calculated with a Kruskal–Wallis one-way analysis of variance.

Significant *p* values are bolded (*p* < 0.05).

Significant differences were found to exist across the deficit groups in age, years of education, and overall aphasia severity. However, the outcome after treatment was modelled independently for each aphasia impairment, limiting the confounding effect of demographic differences between groups.

### Behavioral measures

**Figure 1:**
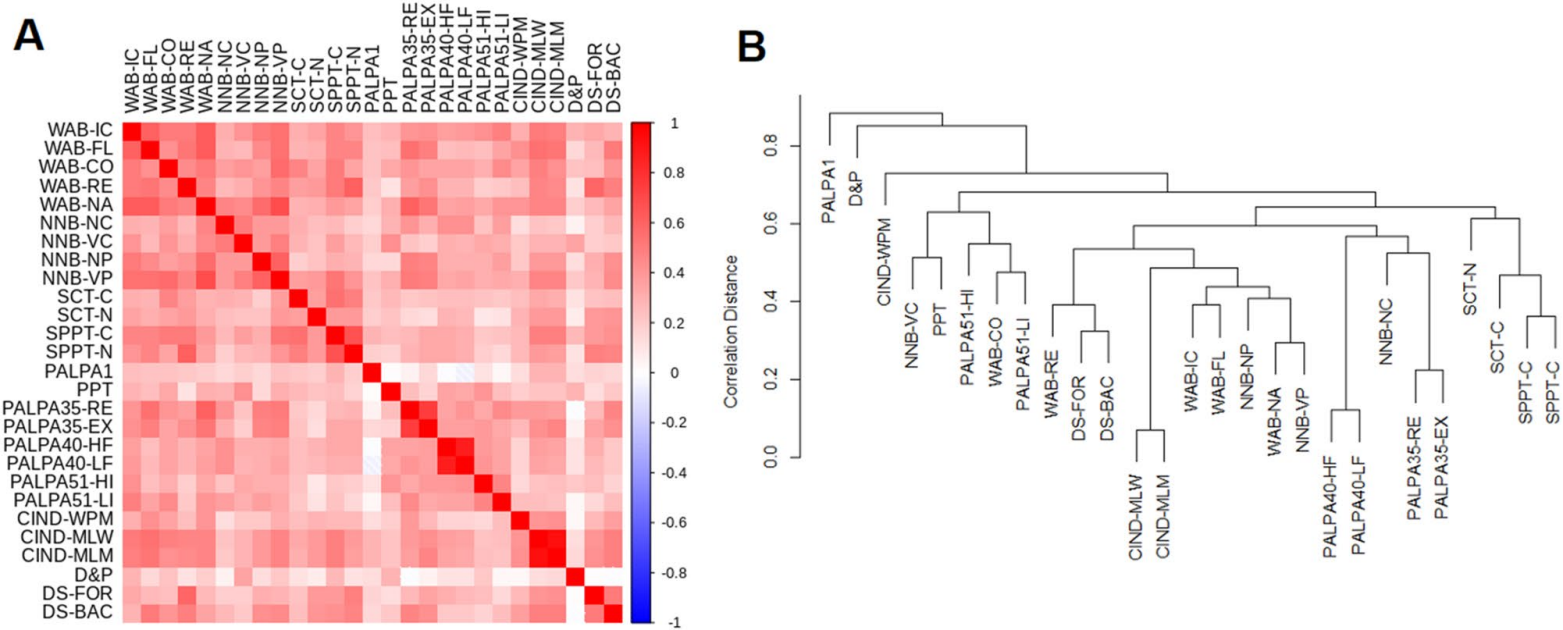
Multicollinearity across Behavioral Measures. (**A**) A shaded color-plot of the correlation matrix across all 27 behavioral measures is shown. Due to imbalance in sample sizes, correlations were first calculated within each impairment group, and then averaged. Box colors correspond to pairwise Kendall’s Tau-b values (red is positive, blue is negative correlation). Only pairwise complete observations were used (no imputation). (**B**) An association dendrogram of behavioral measures is shown. Correlation distance is one minus the absolute pairwise Kendall’s Tau correlation. The dendrogram was created by analyzing correlation distances using hierarchical clustering (Unweighted Pair Group Method with Arithmetic Mean).

Correlations between the behavioral measures included in our aphasia assessment battery are displayed in Fig. 1A. Hierarchical clustering was performed, using one minus the Kendall’s Tau-b correlation as a distance metric between tests (Fig. 1B). Almost all measures correlated positively with all other measures, except for the PALPA 1 and Doors & People measures. These measures clustered independently in the dendrogram. Correlations are especially high within a language measure group (i.e. WAB), and submeasures cluster together. The observed multicollinearity across measures is expected, since nearly all measures test an aspect of language ability.

### Modeling with behavioral measures

**Figure 2:**
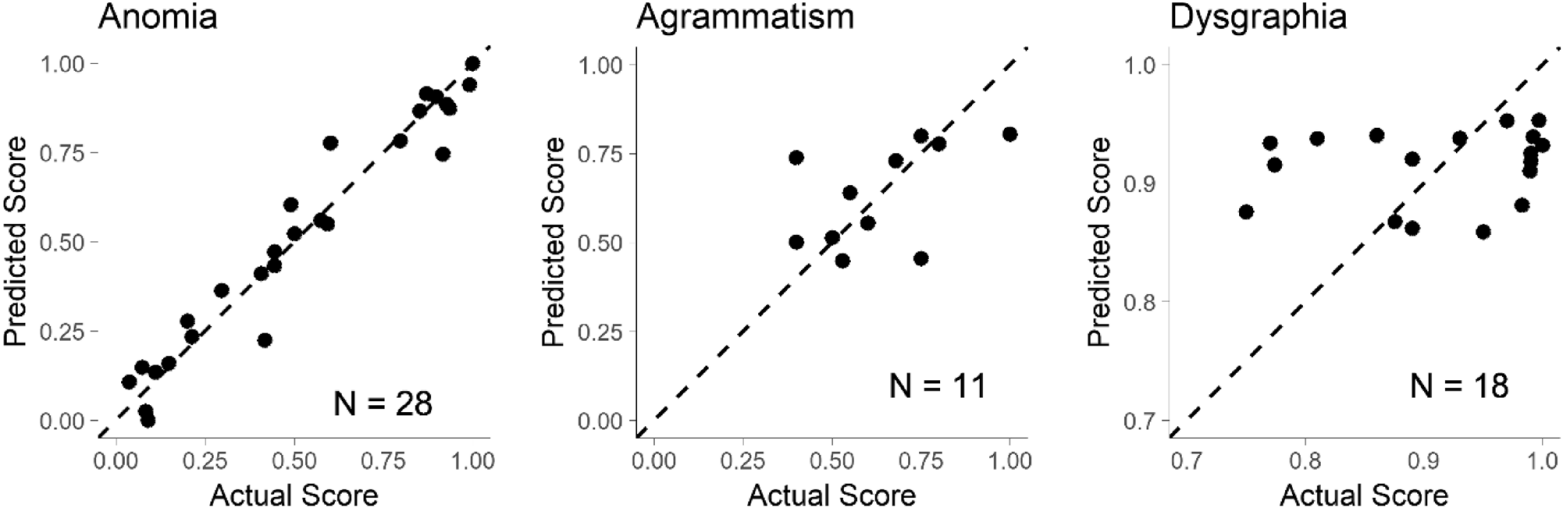
Predicting scores on the post-treatment TSM with behavioral measures. Linear models which predict the TSM after therapy were constructed for each aphasia impairment: anomia, agrammatism, and dysgraphia. The dashed line represents a perfect prediction (predicted score = actual score). Black circles show the median predicted score for each patient across all 1000 imputations during LOOCV.

We predicted performance on the post-treatment TSM using the pre-treatment TSM score as well as all 27 behavioral measures from our aphasia assessment battery (28 predictors per patient). One model was trained on each dataset imputation, and the median output values across all imputations are shown in Fig. 2. The prognostic model for anomia (N = 28) demonstrated low error (MAD = 0.042, 95% CI: 0.018–0.064, *p* < 0.01) and explained much of the variability in outcomes (R^2^ = 0.948, 95% CI: 0.890–0.981, *p* < 0.01). The prognostic model for agrammatism (N = 11) had relatively high error (MAD = 0.089, 95% CI: 0.032–0.207 *p* = 0.232) and inadequately explained the variability in outcomes (R^2^ = 0.257, 95% CI: 0.032–0.800, *p* = 0.111). Similarly, the prognostic model for dysgraphia (N = 18) had relatively high error (MAD = 0.070, 95% CI: 0.041–0.096, *p* = 0.335) and poorly explained the variability in outcomes (R^2^ = 0.029, 95% CI: 0.000–0.308, *p* = 0.495).

Coefficient values and their range across all model imputations are displayed in Table 3. In the anomia model, the highest predictors of positive outcome were the PALPA 35 EXC (0.092), PALPA 40 HF (0.130) and the pre-treatment TSM (0.149), while the highest predictor of negative outcome was the SCT-N (-0.146). Coefficient values were overall well distributed across predictors, with no single predictor exceeding 11% of total coefficient magnitudes. In the agrammatism model, the most impactful predictors of positive outcome were the CIND-WPM (-0.081) and the pre-treatment TSM (0.049), which together represented one quarter of the total coefficient magnitudes. In the dysgraphia model, there were no coefficients which stood out significantly amongst the rest, and the pre-treatment TSM achieved a coefficient of 0.016.

**Table 3:** Coefficients for models using language and cognitive assessments.

Variable	Anomia	Agrammatism	Dysgraphia
WAB-IC	0.014 (0.008, 0.020)	0.006 (0.004, 0.039)	− 0.007 (− 0.008, − 0.006)
WAB-FL	− 0.103 (− 0.106, − 0.099)	0.014 (0.005, 0.045)	0.002 (0.002, 0.003)
WAB-CO	0.010 (− 0.003, 0.013)	− 0.010 (− 0.020, 0.048)	0.005 (0.005, 0.006)
WAB-RE	0.001 (− 0.005, 0.006)	0.030 (0.024, 0.116)	− 0.006 (− 0.006, − 0.006)
WAB-NA	− 0.015 (− 0.021, − 0.001)	− 0.003 (− 0.009, 0.036)	0.002 (0.001, 0.002)
NNB-NC	0.023 (0.015, 0.027)	− 0.010, (− 0.018, 0.025)	− 0.009 (− 0.010, − 0.008)
NNB-VC	− 0.035 (− 0.043, − 0.026)	0.010 (0.004, 0.063)	− 0.007 (− 0.009, − 0.006)
NNB-NP	− 0.009 (− 0.012, 0.007)	0.006 (0.003, 0.056)	0.020 (0.019, 0.020)
NNB-VP	− 0.029 (− 0.040, − 0.017)	0.019 (0.014, 0.087)	0.021 (0.020, 0.024)
SCT-C	0.027 (0.023, 0.029)	− 0.015 (− 0.025, − 0.003)	0.013 (0.012, 0.013)
SCT-N	− 0.146 (− 0.151, − 0.137)	− 0.004 (− 0.006, 0.059)	− 0.003 (− 0.004, − 0.003)
SPPT-C	0.037 (0.032, 0.042)	0.015 (0.011, 0.109)	0.022 (0.021, 0.023)
SPPT-N	− 0.081 (− 0.084, − 0.078)	− 0.023 (− 0.026, 0.045)	0.020 (0.019, 0.020)
PALPA1	0.041 (0.036, 0.048)	0.018 (0.009, 0.039)	0.029 (0.028, 0.029)
PPT	0.037 (0.035, 0.039)	0.026 (0.019,0.044)	0.009 (0.009, 0.010)
PALPA35-RE	0.057 (0.051, 0.062)	0.001 (− 0.005, 0.058)	0.020 (0.017, 0.021)
PALPA35-EX	0.092 (0.096, 0.098)	0.027 (0.021, 0.109)	0.027 (0.023, 0.028)
PALPA40-HF	0.130, (0.122, 0.137)	0.009 (− 0.001, 0.094)	0.024 (0.023, 0.024)
PALPA40-LF	− 0.034 (− 0.038, − 0.032)	− 0.026 (− 0.033, 0.048)	0.017 (0.017, 0.018)
PALPA51-HI	0.054 (0.050, 0.056)	0.035 (0.030, 0.060)	0.016 (0.016, 0.017)
PALPA51-LI	0.009 (0.006, 0.013)	− 0.031 (− 0.041, − 0.021)	0.014 (0.014, 0.015)
CIND-WPM	− 0.015 (− 0.022, − 0.006)	− 0.081 (− 0.107, − 0.074)	− 0.005 (− 0.009, − 0.002)
CIND-MLW	0.030 (0.009, 0.052)	0.018 (0.014, 0.073)	0.010 (0.008, 0.013)
CIND-MLM	− 0.007 (− 0.017, 0.034)	0.016 (0.013, 0.077)	0.005 (0.002, 0.007)
D&P	− 0.032, (− 0.037, − 0.029)	− 0.002 (− 0.016, 0.036)	0.021 (0.020, 0.021)
DS-FOR	0.007 (− 0.003, 0.010)	0.028 (0.027, 0.096)	− 0.007, (− 0.008, − 0.006)
DS-BAC	0.082 (0.072, 0.085)	− 0.006 (− 0.016, 0.073)	0.010 (0.009, 0.012)
TSM	0.149 (0.129, 0.154)	0.049 (0.045, 0.126)	0.016 (0.016, 0.017)

The median model coefficient for each behavioral model is shown, followed by the range of minimum and maximum coefficients across 1000 data imputations.

### Modeling with GICA fALFF

**Figure 3:**
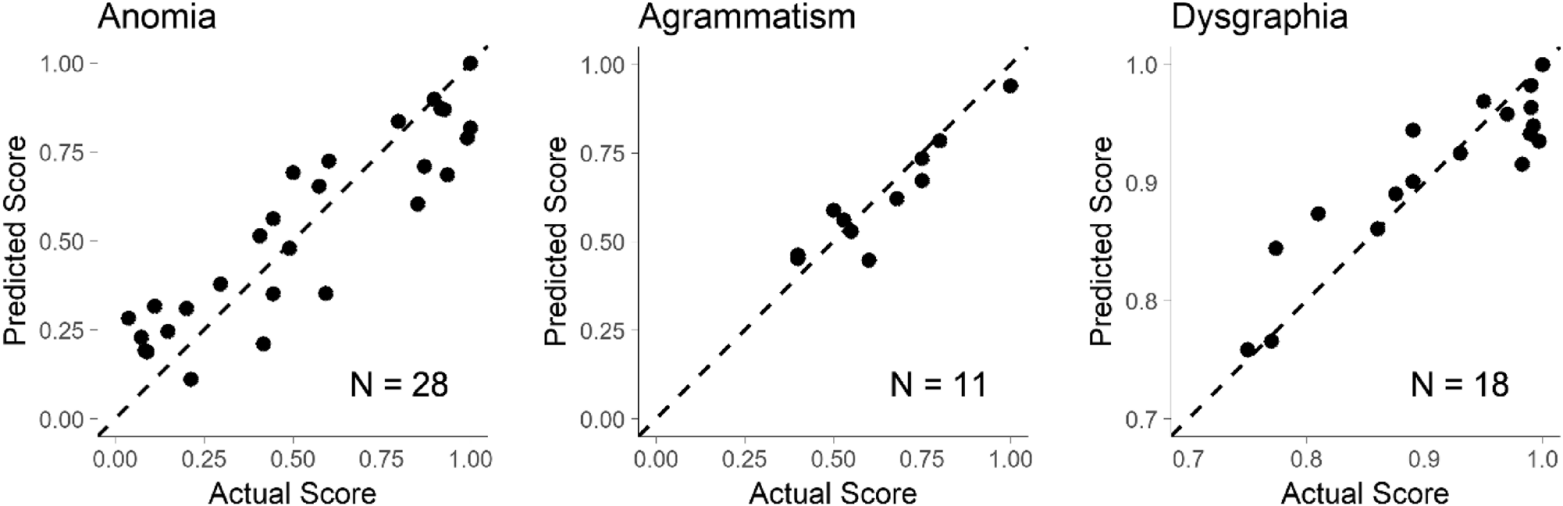
Predicting scores on the post-treatment TSM with GICA fALFF and pre-treatment TSM. Linear models which predict the TSM after therapy were constructed for each aphasia impairment (anomia, agrammatism, and dysgraphia) using a combination of the pre-treatment TSM and the fALFF for each GICA component. The dashed line represents a perfect prediction (predicted score = actual score). Black circles show the predicted score for each patient using LOOCV. For the agrammatism model, the circles represent median values across 1000 imputations.

We next built prognostic models using fALFF values of independent components and baseline aphasia severity as measured by the pre-treatment TSM (Fig. 3). These models did not contain any of the 27 additional behavioral measures used in the above models. The prognostic model for anomia (N = 28) again demonstrated low error (MAD = 0.109, 95% CI: 0.095–0.172, *p* < 0.01) and explained much of the variability in outcomes (R^2^ = 0.816, 95% CI: 0.698–0.900, *p* < 0.01). The prognostic model for agrammatism (N = 11) also had low error (MAD = 0.051, 95% CI: 0.016–0.095, *p* = 0.012) and explained much of the variability in outcomes (R^2^ = 0.876, 95% CI: 0.402–0.992, *p* < 0.01). The prognostic model for dysgraphia (N = 18) had low error (MAD = 0.017, 95% CI: 0.008–0.051, *p* < 0.01) and explained much of the variability in outcomes (R^2^ = 0.822, 95% CI: 0.621–0.922, *p* < 0.01).

Coefficients for each model are displayed in Table 4. The largest coefficients in the anomia model were due to the pre-treatment TSM (0.272) and fALFF of Component 18 (0.132). In the agrammatism model, the coefficients for fALFF of Component 5 (0.062), fALFF of Component 1 (0.057), and the pre-treatment TSM (0.037) were largest. Coefficients in the dysgraphia model were overall more evenly distributed, with only the fALFF of Component 19 (0.080) standing out.

**Table 4:** Coefficients for models using baseline severity and component fALFF.

Variable	Anomia	Agrammatism	Dysgraphia
fALFF 1	0.024	0.057 (0.039, 0.074)	− 0.028
fALFF 2	− 0.016	0.013 (− 0.002, 0.028)	0.007
fALFF 3	− 0.090	− 0.022 (− 0.025, − 0.017)	0.019
fALFF 4	0.022	− 0.008 (− 0.009, − 0.006)	− 0.036
fALFF 5	0.072	− 0.062 (− 0.071, − 0.053)	− 0.008
fALFF 6	− 0.035	0.027 (0.016, 0.041)	− 0.009
fALFF 7	0.008	0.004 (0.003, 0.005)	0.001
fALFF 8	0.008	− 0.014 (− 0.014, − 0.013)	− 0.009
fALFF 9	− 0.038	0.001 (− 0.019, 0.021)	− 0.033
fALFF 10	− 0.038	0.012 (− 0.005, 0.030)	0.027
fALFF 11	− 0.055	− 0.030 (− 0.036, − 0.026)	0.008
fALFF 12	0.062	− 0.023 (− 0.044, 0.000)	0.000
fALFF 13	0.026	− 0.031 (− 0.039, − 0.024)	0.034
fALFF 14	0.003	0.030 (0.020, 0.038)	− 0.001
fALFF 15	− 0.069	0.006 (0.003, 0.011)	− 0.023
fALFF 16	− 0.042	0.035 (0.030, 0.040)	− 0.047
fALFF 17	− 0.020	− 0.013 (− 0.033, 0.003)	0.029
fALFF 18	0.132	0.031 (0.005, 0.061)	0.017
fALFF 19	0.029	− 0.011 (− 0.015, − 0.005)	0.080
fALFF 20	0.004	− 0.002 (− 0.009, 0.007)	− 0.038
TSM	0.272	0.037 (0.033, 0.043)	0.003

The median model coefficient for each behavioral model is shown. For the agrammatism model, the range of minimum and maximum coefficients is shown across 1000 data imputations. The anomia and dysgraphia models had no missing data for this analysis, so no imputations were computed for the corresponding groups.

## Discussion

### Behavioral assessment multicollinearity

We have found strong multicollinearity across behavioral measures (Fig. 1), which has been observed previously^62^. All behavioral measures show a consistent weak correlation with nearly all other measures (median pairwise correlation of 0.36), suggesting that there is substantial overlap in the information being collected across assessments. In addition, measures from within the same assessment have even higher association, such as in the PALPA 40 where the correlation in spelling scores between high-frequency and low-frequency words is 0.88. This is significant because comprehensive aphasia testing is laborious for both patients and practitioners, and there is a need for shorter-form aphasia probes that can deliver adequate assessments^63^. The quick aphasia battery is a potential solution which can be rapidly administered and has high correlation with corresponding WAB measures^41^. Our analysis shows that there is high correlation between WAB measures, suggesting that further efficiency gains in clinical aphasia assessment may be realized by prioritizing orthogonal measures.

While multicollinearity is strongest between language assessments, there is also some correlation with the Digit Span, an assessment of verbal short-term memory. Relatively few language assessments correlate with the Doors & People assessment, which is also a verbal memory assessment (median pairwise correlation of 0.13). However, Doors & People correlates weakly with the WAB Information Content measure, which has one of the highest median correlations with all other assessments (0.43). It is therefore possible that there is some unique information shared between the D&P and the WAB-IC measures that is not shared between the WAB-IC and most other language assessments. These observations support the current understanding that post-stroke aphasia is a multidimensional disorder that may present with a range of language and cognitive impairments as a result of damage to multiple brain areas^19,64^.

### Model performance

Prognostic models using initial severity and behavioral measures performed best in the anomia group. The behavioral model for anomia relied most on the pre-treatment TSM, PALPA 35 EXC, and PALPA 40 HF measures, suggesting that pre-treatment reading and spelling ability were approximately as important as initial anomia severity in prognosis. This is not entirely surprising, as patients who perform better on nonspecific language assessments may have more intact language networks prior to therapy. Patients with improved baseline language function will tend to score higher than those with more impaired baseline language function, even after therapy. This may help explain why the majority of coefficients across the behavioral models were positive. However, higher initial performance on some language metrics indicated poorer outcome (i.e. SCT-N and SPPT-N in the anomia model). There is a pattern in the anomia model where paired measures have opposite coefficient signs (i.e. SCT-N and SCT-C) indicating that anticorrelated performance on measures which are typically correlated may carry some prognostic information. However, it is challenging to derive strong conclusions about individual predictor variables based on model coefficients due to the interactions between model regularization and multicollinearity. Ridge (L2-norm) regularization will incentivize the model to distribute coefficient magnitude across predictor variables during training. When multicollinearity is present, there is relatively more shared information between predictor variables, so model coefficients tend to be small even if some predictor variables are strong individual predictors.

Prognostic models using initial severity and GICA fALFF activity performed best in the agrammatism and dysgraphia groups. While the anomia model demonstrated strong performance as well, much of this can be attributed to the pre-treatment TSM, further suggesting that behavioral assessments were more valuable than rsfMRI in the anomia group. As GICA selects components to be statistically independent of one another, fALFF values are not multicollinear and have clearer interpretation of model coefficients when regularization is used. The GICA component maps generated in this analysis do not resemble known language networks, but rather demonstrate strong weighting in the ventricles and brain regions known to be sensitive to physiological motion. This reflects our minimal image preprocessing pipeline and inclusion of all voxels within the brain. When analysis is repeated using rsfMRI data that was filtered to remove cardiac-driven physiological motion, the predictive power of the models is lost. We hypothesize that the data-driven patterns picked up by our GICA components and the fALFF-based prognostic models are functioning as proxy variables for regional cerebral blood flow (rCBF).

Arterial spin labeling studies have shown that the patterns of cerebral blood flow are known to be aberrant in both the acute and chronic phases of stroke recovery^65,66^. Alterations in regional cerebral blood flow (rCBF) have been tied to cognitive functioning in numerous neuropsychiatric conditions such as Alzheimer’s Disease, frontotemporal dementia, adolescent ADHD, and schizophrenia^67–70^. In post-stroke aphasia, one study has shown that low-frequency repetitive transcranial magnetic stimulation (LF-rTMS) drives an increase in local rCBF that is proportional to the degree of language recovery^71^. Furthermore, the areas which experienced changes in rCBF extended beyond where LF-rTMS was applied. In our models, the coefficients are overall well distributed and no one component explains the majority of variance in outcomes. It is possible that global patterns of aberrant rCBF are valuable in predicting response to SLT in chronic post-stroke aphasia.

### Limitations

There were several limitations present in this study. First, we encountered challenges in model selection due to sample size. While we recognize that the relationship of baseline aphasia severity to treatment outcomes likely has nonlinear components, we were limited to linear models with heavy regularization to overcome the challenge of having more predictor variables than subjects. Furthermore, model validation would have benefited from an independent validation set, however this would have strained training further to where LOOCV was the most viable approach. Recruitment was limited in part by the extensive imaging and behavioral testing that was performed for each subject alongside hours of treatment. Second, while subjects were admitted to the study based on a singular aphasia impairment (anomia, agrammatism, or dysgraphia), this design was based on treatment assignment and did not take into account the possibility of overlapping impairment and any possible nonlinear effects treatment. Third, while we model responses to three treatment protocols, there are many other protocols for aphasia therapy. We have demonstrated large variability in model performance across protocols, and this variability likely extends to protocols not examined here. Fourth, while we model performance on the TSM after treatment as our primary outcome, it may be more precise to instead model the individual change in TSM performance instead (post-treatment TSM—pre-treatment TSM). However, we were constrained by ceiling effects where subjects with high baseline performance did not have substantial room for improvement due to the dynamic range of the assessments used. When individual performance changes are considered, patients with known positive prognostic factors (i.e. high baseline performance) are interpreted during training as experiencing little performance gain. This convolutes model training leading to decreased performance, as our linear model lacks the capacity to condition the effects of other favorable prognostic variables on baseline severity (a nonlinear interaction).

## Conclusions

High-performance predictive models of individual response to therapy were trained for each aphasia impairment. Models based on GICA fALFF were overall higher performing and more consistent than models based on behavioral measures alone. Furthermore, the average performance of the GICA fALFF models (R^2^ = 0.816–0.876) is competitive when compared to prior work modelling aphasia outcomes (R^2^ = 0.56, 0.73) which have relied on behavioral or anatomical variables^16,17^. This encourages further study of rsfMRI as a prognostic tool for post-stroke aphasia. Continued effort on developing prognostic models which estimate treatment response trajectories may ultimately improve treatment. A series of high-performance prognostic models could be used to estimate the distribution of outcomes for a variety of therapeutic options, opening the door to personalized treatment. This approach may help overcome the unexplained variability in response to aphasia treatment, giving patients and practitioners more agency in the treatment selection process.

rsfMRI has several advantages over conventional language measures in assessment and prognosis. Language assessments have a limited dynamic range and therefore a limit of deficit severity to which they are sensitive. This could make assessment of treatment response difficult in patients with near the assessment ceiling or floor, and this challenge was observed in our dataset. In contrast, features based on rsfMRI are more continuous and may have higher dynamic range, offering potential to equitably assess a wider range of aphasia severity. Creating quantitative profiles of aphasia severity from rsfMRI may be easier in practice than refining a battery of existing language measures due to the high dimensionality of rsfMRI data. However, there are challenges facing clinical adoption of rsfMRI for aphasia. Imaging is relatively expensive, and accessibility to quality scanning varies across patient populations. It is challenging to image patients who are claustrophobic or unable to keep still. Clinical adoption of rsfMRI may become feasible with further advancements in imaging technology and/or support from healthcare systems.

## Supplementary Information

Supplementary Information

## Data Availability

The minimal dataset needed to interpret, replicate, and build on the findings reported in this paper are available from the corresponding author on reasonable request.

## Code availability

Source code for the methods applied in this work is available at https://github.com/miorga7 (repository: aphasia_prediction, R code).
