# Supplementary material for: Predicting language recovery in post-stroke aphasia using behavior and functional MRI
Source: Sci Rep. 2021 Apr 19;11:8419. doi: 10.1038/s41598-021-88022-z (PMC8055660; doi:10.1038/s41598-021-88022-z)
Supplement: Supplementary file 1 — Supplementary Information [file 41598_2021_88022_MOESM1_ESM.pdf]

Supplementary Information

Predicting Language Recovery in Post-Stroke Aphasia using Behavior and Functional MRI

Michael Iorga*[1-3], James Higgins[1,3], David Caplan[1,4], Richard Zinbarg[1,5], Swathi Kiran[1,6], Cynthia K. Thompson[1,7,8], Brenda Rapp[1,9], Todd B. Parrish[1,3]

[1] Center for the Neurobiology of Language Recovery, Northwestern University, Evanston, IL, USA
[2] Department of Biomedical Engineering, McCormick School of Engineering, Northwestern University, Chicago, IL, USA
[3] Department of Radiology, Feinberg School of Medicine, Northwestern University, Chicago, IL, USA
[4] Department of Neurology, Massachusetts General Hospital, Harvard Medical School, Boston, MA, USA
[5] Department of Psychology, Northwestern University, Evanston, IL, USA
[6] Department of Speech, Language, and Hearing, College of Health & Rehabilitation, Boston University, Boston, MA, USA
[7] Department of Communication Sciences and Disorders, School of Communication, Northwestern University, Evanston, IL, USA
[8] Department of Neurology, Neurology, Feinberg School of Medicine, Northwestern University, Chicago, IL, USA
[9] Department of Cognitive Science, Krieger School of Arts & Sciences, Johns Hopkins University, Baltimore, MD, USA

*corresponding author

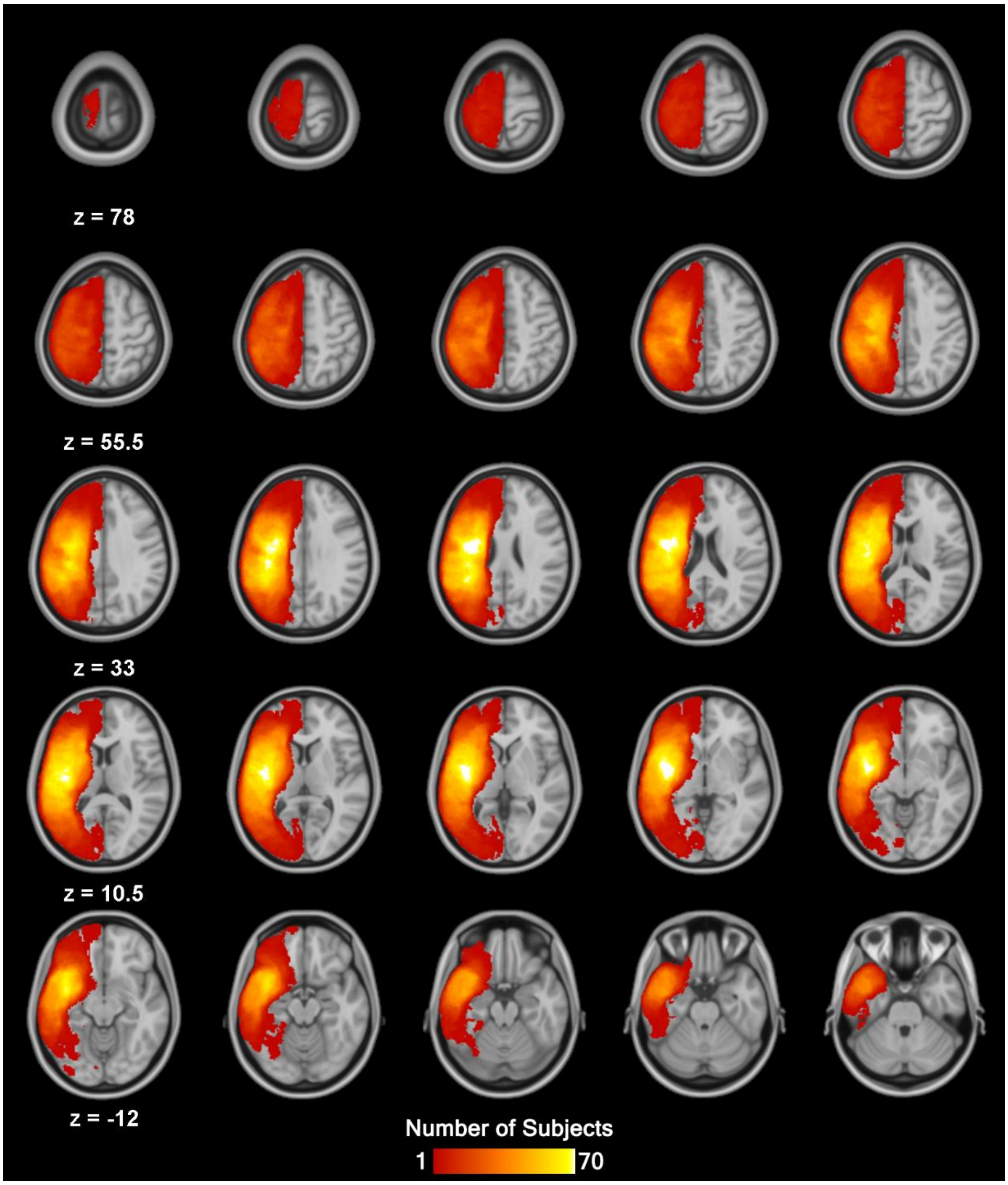

Supplemental Figure S1: Composite map of participant brain lesions. Brain lesions were manually traced by trained operators on the T1 images using freeview (Freesurfer) and MRIcro. During tracing, the brain was inspected in three orthogonal views simultaneously to improve lesion detection. The final masks were reviewed, revised, and approved by several researchers.

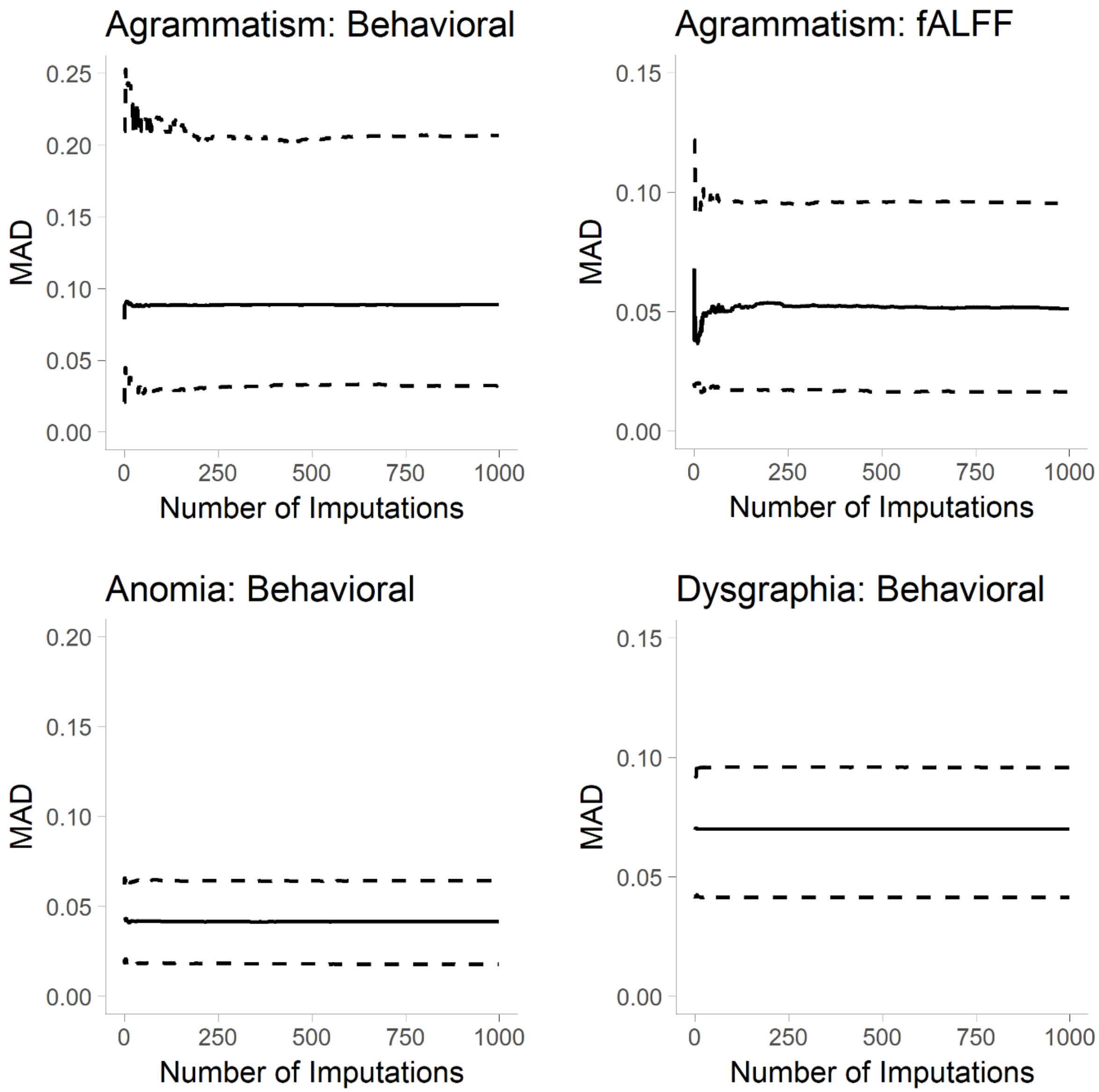

Supplemental Figure S2: Median Correlation Convergence Plot. Median correlations across imputations (solid line) along with bootstrapped 95% confidence intervals (dashed lines) are displayed for regression analyses with missing data: agrammatism behavioral model (top left), agrammatism fALFF model (top right), anomia behavioral model (bottom left), and dysgrampia behavioral model (bottom right). Median correlation stabilized in all cases across 1000 imputations.

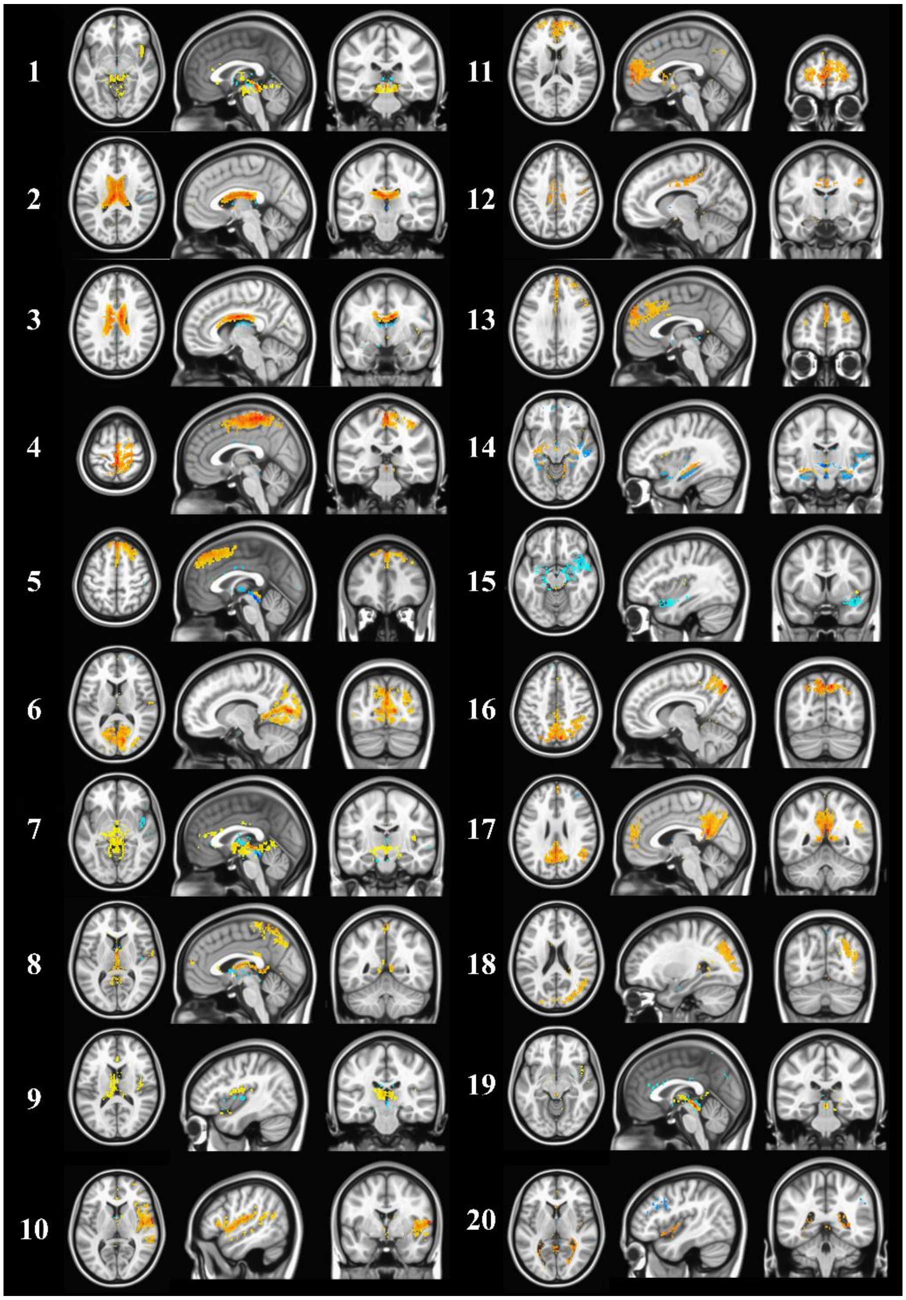

Supplemental Figure S3: Aggregate GICA Components. Backprojected spatial components were averaged across subjects to create aggregate component maps (shown). Sagittal, axial, transverse planes are displayed about each component's peak point. Color corresponds to the z-score of the voxel coefficient (1-sided t test). Colors are scaled to each component's range ($3 \leq |z| \leq |z_{peak}|$). Red voxels are component correlated while blue voxels are component anticorrelated.
